# Ellipticine and benzo(a)pyrene increase their own metabolic activation via modulation of expression and enzymatic activity of cytochromes P450 1A1 and 1A2

**DOI:** 10.2478/V10102-010-0033-z

**Published:** 2010-11

**Authors:** Dagmar Aimová, Jitka Poljaková, Věra Kotrbová, Michaela Moserová, Eva Frei, Volker M. Arlt, Marie Stiborová

**Affiliations:** 1 Department of Biochemistry, Faculty of Science, Charles University, Prague, Albertov 2030, 128 40 Prague 2, CZECH REPUBLIC; 2 Division of Molecular Toxicology, German Cancer Research Center, Im Neuenheimer Feld 280, 69120 Heidelberg, GERMANY; 3 Section of Molecular Carcinogenesis, Institute of Cancer Research, Brookes Lawley Building, Sutton, Surrey SM2 5NG, UNITED KINGDOM

**Keywords:** benzo(a)pyrene, ellipticine, induction, cytochromes P450, NADPH:cytochrome P450 reductase, HRN™ mice

## Abstract

Two compounds known to covalently bind to DNA after their activation with cytochromes P450 (CYPs), carcinogenic benzo(a)pyrene (BaP) and an antineoplastic agent ellipticine, were investigated for their potential to induce CYP and NADPH:CYP reductase (POR) enzymes in rodent livers, the main target organ for DNA adduct formation. Two animal models were used in the study: (i) rats as animals mimicking the fate of ellipticine in humans and (ii) mice, especially wild-type (WT) and hepatic POR null (HRN™) mouse lines. Ellipticine and BaP induce expression of CYP1A enzymes in livers of experimental models, which leads to increase in their enzymatic activity. In addition, both compounds are capable of generating DNA adducts, predominantly in livers of studied organisms. As determined by ^32^P postlabelling analysis, levels of ellipticine-derived DNA adducts formed *in vivo* in the livers of HRN™ mice were reduced (by up to 65%) relative to levels in WT mice, indicating that POR mediated CYP enzyme activity is important for the activation of ellipticine. In contrast to these results, 6.4 fold higher DNA binding of BaP was observed in the livers of HRN™ mice than in WT mice. This finding suggests a detoxication role of CYP1A in BaP metabolism *in vivo*. In *in vitro* experiments, DNA adduct formation in calf thymus DNA was up to 25 fold higher in incubations of ellipticine or BaP with microsomes from pretreated animals than with controls. This stimulation effect was attributed to induction of CYP1A1/2 enzymes, which are responsible for oxidative activation of both compounds to the metabolites generating major DNA adducts *in vitro*. Taken together, these results demonstrate that by inducing CYP1A1/2, ellipticine and BaP modulate their own enzymatic metabolic activation and detoxication, thereby modulating their either pharmacological (ellipticine) and/or genotoxic potential (both compounds).

## Introduction

Ellipticine and benzo[*a*]pyrene (BaP) are compounds exhibiting significant biological activities. Ellipticine is an efficient anticancer agent (for a summary, see Stiborová *et al.*, 2006b), while BaP is a strong carcinogen (for a summary, see Arlt *et al.*, 2008). Therefore, both two compounds were employed by us for studies concerning their phatmacological and toxicological effects.

**Figure 1:**
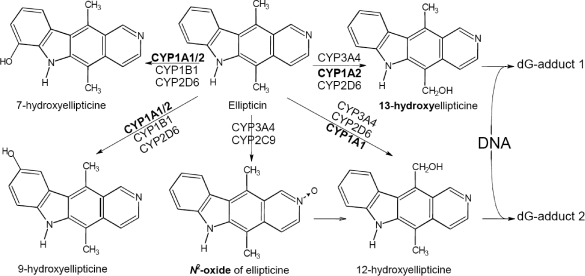
Metabolism and activation of ellipticine (5,11-dimethyl-6*H*-pyrido[4,3-*b*]carbazole) by cytochromes P450.

Ellipticine, an alkaloid isolated from *Apocyanaceae* plants, and its derivatives exhibits significant antitumor and anti-HIV activities, characterized by high efficiencies against several types of cancer and rather limited toxic side effects, including complete lack of hematological toxicity. Nevertheless, ellipticines are potent mutagens. Several mechanisms of their antitumor, mutagenic and cytotoxic activities have been hitherto suggested: (i)intercalation into DNA; (ii)inhibition of DNA topoisomerase II activity; (iii) selective inhibition of p53 protein phosphorylation; (iv) disruption of the energy balance of cells by uncoupling mitochondrial oxidative phosphorylation (for a summary, see Stiborová *et al.*, 2001; 2006b). Recently, we have shown that ellipticine also binds covalently to DNA *in vitro* and *in vivo,* after being enzymatically activated with cytochromes P450 (CYP) (Figure 1) or peroxidases, suggesting a third possible mechanism of action (Stiborová *et al.*, 2001; 2003a, b; 2004; 2007a).

On the basis of *in vitro* studies, human and rat CYPs of 1A and 3A subfamilies seem to be the predominant enzymes oxidizing ellipticine either to metabolites that are excreted (7- or 9-hydroxyellipticine) or form DNA adducts (12- or 13-hydroxy-ellipticine) (Stiborová *et al.*, 2001; 2003b; 2004; 2006; Kotrbová *et al.*, 2006). Besides these CYPs, peroxidases such as mammalian cyclooxygenases (COX-1 and -2), lactoperoxidase and myeloperoxidase, efficiently generate the same ellipticine-derived DNA adducts *in vitro* (Stiborová *et al.*, 2007a; Poljaková *et al.*, 2006). Identical DNA adducts were also detected in cells in culture, in which both CYPs and peroxidases are expressed, such as human breast adenocarcinoma MCF-7 cells (Boìek-Dohalská *et al.*, 2004), leukemia HL-60 and CCRF-CEM cells (Poljaková *et al.*, 2007) and V79 Chinese hamster lung fibroblasts transfected with human CYP3A4, 1A1 and 1A2 (Frei *et al.*, 2002). After *i.p*. administration of ellipticine, the ellipticine-DNA adduct levels seem to be related to CYP3A1 and 1A content in different tissues of rat, but the real impact of CYPs or peroxidases in this process could not be still exactly evaluated (Stiborová *et al.*, 2003a, 2007b).

**Figure 2:**
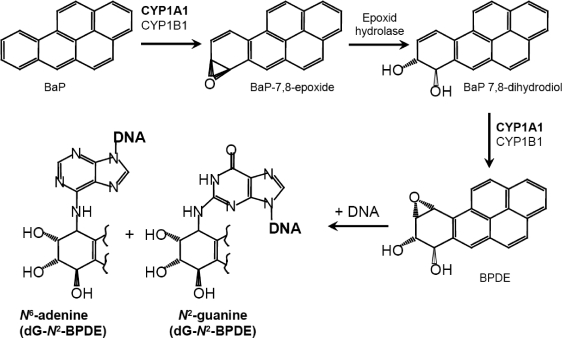
Metabolic activation and DNA adduct formation by benzo(a)pyrene: The typical 3-step activation process with contribution of CYP1A1 or CYP1B1 and epoxide hydrolase leads to the formation of the ultimately reactive species, benzo[a]pyrene-7,8-dihydrodiol-9,10-epoxide (BPDE) that can react with DNA, forming adducts preferentially at guanine residues.

BaP, as the second model compound in this comparative study is, similarly to the other polycyclic aromatic hydrocarbons (PAHs), mutagenic and carcinogenic (IARC, 1983; Phillips 1999, 2002). PAHs are produced mainly by incomplete combustion or pyrolysis of organic matter and are ubiquitous in the environment, leading to measurable background levels of exposure in the general population (IARC, 1983). Beside the inhalation of polluted air, the main routes of exposure are through tobacco smoke, diet (Phillips 1999, 2002) and occupational exposition throughout e.g. coal, coke or coal-tar processing and use of coal-tar products (IARC, 1983). Prior to the reaction with DNA, BaP analogously to ellipticine requires metabolic activation (Figure 2), which is an essential step in the mechanism by which BaP exerts its genotoxic effects. CYP1A1 and CYP1B1 are widely accepted to be the most important enzymes in the metabolic activation of BaP (Baird *et al*., 2005). However, current studies show that BaP-induced DNA damage was increased in mice lacking CYP1A1, indicating that *in vivo* the CYP1A1 enzyme plays a detoxification role, and protects mice against BaP toxicity (Uno *et al*., 2004, 2006). PAHs affect the expression of numerous enzymes involved in metabolism of xenobiotics (including CYP1A1) mainly *via* the aromatic hydrocarbon receptor (AhR). AhR-dependent inducibility was correlated to the predisposion to some types of cancer (Kouri *et al.*, 1973).

The detailed knowledge on the role of CYP enzymes in activation and/or detoxication of BaP and ellipticine and that on their induction mediated by these xenobiotics, is crucial for the possibility to modify their carcinogenic and/or the therapeutic efficiency. Therefore, this field was extensively investigated in our laboratory. To investigate the real role of CYPs in metabolism of both compounds, we have used several animal models, such as rats, rabbits and/or mice in our previous studies (Stiborová *et al.*, 2001; 2003a; b; 2004; 2006; 2007b; 2008; Kotrbová *et al*., 2006; Arlt *et al*., 2008). In the case of mice, the HRN™ (Hepatic Cytochrome P450 Reductase Null) mice, the mouse line lacking the hepatic NADPH:CYP oxidoreductase (POR), the unique electron donor to CYPs, which results in the loss of essentially all hepatic CYP function (Henderson *et al.*, 2003, 2006), was utilized (Stiborová *et al.*, 2008; Arlt *et al*., 2008).

Here, we summarize the results obtained with rats and mice previously, and present novel data obtained with these animal models. Such a study is necessary to evaluate results found till the present time and to suggest which further studies are necessary to improve our knowledge in this field.

## Material and Methods

### Animal models

The study was conducted in accordance with the Regulations for the Care and Use of Laboratory Animals (311/1997, Ministry of Agriculture, Czech Republic), which complies with Declaration of Helsinki. Rats, the animal model found to be suitable to mimic the fate of ellipticine in humans (Stiborová *et al.*, 2003a; 2006), and two mouse lines, namely,.(i) „Hepatic Reductase Null“ (HRN™) mice based on a C57BL/6 background (CXR Bioscience Ltd, Dundee, UK), in which NADPH:cytochrome P450 reductase (POR) is specifically deleted in the liver (*Por* ^lox/lox^ + *Cre* ^ALB^) (Henderson *et al.*, 2003, 2006) and (ii) mice homozygous for loxP sites at the Por locus (Por^lox/lox^) as wild-type (WT) mice, were used in this study.

### Treatment of animals with ellipticine and benzo(a)pyrene

Male and female Wistar rats (~100 g) were treated with a single dose of 4, 40 or 80 mg/kg body weight (n = 3) of ellipticine by intraperitoneal injection as described (Aimová *et al*., 2007). Ellipticine was dissolved in sunflower oil/dimethyl sulphoxide (1:1, v/v) at a concentration of 6 mg/ml, control animals (n = 3) received solvent only. The doses of ellipticine used for the treatment of rats are in the range of dosage in human therapy (80–100 mg/m^2^).

Groups (*n*=3) of female HRN™ and WT mice (3 months old, 25–30 g) were treated intraperitoneally with a single dose of 10 mg ellipticine per kg body weight as described previously (Stiborová *et al.*, 2008). Ellipticine was administered as 10 mg/ml solution in distilled 1% acetic acid, control animals (n = 3) received solvent only.

To evaluate the BaP-mediated induction of CYP1A, groups (n = 3) of HRN™ and WT female mice were treated with 125 mg BaP per kg body weight once daily for five days by intraperitoneal injection. BaP was dissolved in corn-oil at a concentration of 12.5 mg/ml, control animals (n = 3) received solvent only (Arlt *et al*., 2008).

### Preparation of microsomes and assays

Microsomes were isolated from pooled rodent livers as described (Stiborová *et al.*, 2003b). Protein concentrations in the microsomal fractions (bicinchoninic acid protein assay with bovine serum albumin as a standard), the activities of hepatic microsomal CYP1A1/2 (7-ethoxyresorufin *O*-deethylation, EROD) and POR (using cytochrome c) as well as the protein levels of these enzymes (Western Blot) were determined as described previously (Stiborová *et al.*, 2003a, b, 2005).

Oxidation of ellipticine by hepatic microsomes. Incubation mixtures contained 50 mM potassium phosphate buffer (*pH*7.4), NADPH-generating system (1 mM NADP+, 10 mM D-glucose 6-phosphate, 1 U/ml D-glucose 6-phosphate dehydrogenase), 0.2 mg microsomal protein, 10 µM ellipticine (dissolved in 5 µl methanol) in a final volume of 500 µl. The reaction was initiated by adding the substrate. After incubation in open glass tubes (37°C, 20 min) the reaction was stopped by adding 100 µl of 2 M NaOH, 5 µl of 1 mM phenacetine in methanol was added as an internal standard. Ellipticine metabolites were extracted twice with ethyl acetate (2×1 ml) and analyzed by HPLC as described (Stiborová *et al.*, 2006; 200ß).

Activation of ellipticine or BaP by hepatic microsomes

Incubation mixtures (final volume of 750 µl) used to asses DNA adduct formation consisted of 50 mM potassium phosphate buffer (pH7.4), 1mM NADPH, 0.5 mg of microsomal proteins and 0.5 mg of calf thymus DNA. Incubations were also carried out in the presence of a COX cofactor, 0.1mM arachidonic acid instead of NADPH, and additionally 5 mM magnesium chloride. The reaction was initiated by adding 0.1 mM ellipticine (dissolved in 7.5 µl methanol) or 0.1 mM BaP (dissolved in 7.5 µl dimethyl sulphoxide). Incubations at 37°C were carried out for 30 or 90 min with ellipticine or BaP, respectivelly. DNA was isolated from the residual water phase by the phenol/chloroform extraction method as described (Stiborová *et al.*, 2001).

### Inhibition studies

The following chemicals were used to inhibit the activation of ellipticine and BaP by mouse hepatic microsomes: α-naphthoflavone (α-NF), which inhibits CYP1A1 and 1A2; indomethacin, a selective inhibitor of COX; α-lipoic acid (α-LA), which inhibits POR; ellipticine, frequently utilized as competitive inhibitor of CYP1A1 enzyme. Inhibitors were added to incubation mixture in 7.5 µl of methanol to yield final concentrations of 0.1mM and pre-incubated at 37°C for 10 min with NAD*PH* prior to adding substrate (ellipticine or BaP) nd then incubated for as described above. After the incubation, DNA was isolated as described above.

### Measurement of DNA adducts

^32^P-postlabeling analysis with nuclease P1 enrichment, thin-layer chromatography (TLC) and high performance liquid chromatography (HPLC) of ^32^P-labelled 3′5′-deoxyribonucleoside bisphosphate adducts with ellipticine were done as reported recently (Stiborová *et al*., 2001; 2003a; 2004; 2007a). DNA adducts formed by BaP were analyzed analogously, using the ^32^P-postlabeling technique as described previously (Arlt *et al*., 2008).

## Results

### Induction of hepatic CYP1A by ellipticine and BaP

**Figure 3:**
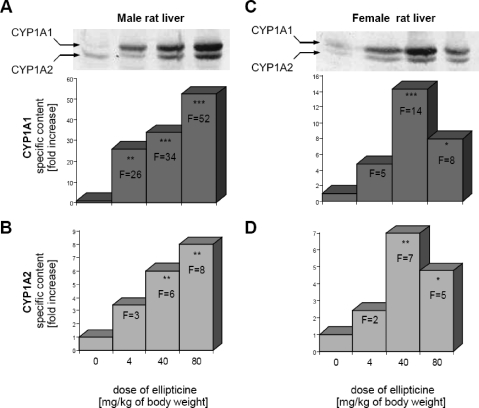
Induction of CYP1A1 (**A,C**) and 1A2 (**B,D**) proteins in livers of male (**A,B**) and female (**C,D**) rats, uninduced or treated with 4, 40 or 80 mg of ellipticine per kg of body weight. Inset in A and C: immunoblots of microsomal CYP1A1 and 1A2 from untreated and ellipticine-treated male and female rats, respectively, stained with antibody against rat CYP1A1. Mean values shown in figure represent results obtained from livers of three rats (n = 3), SD<15%. Values significantly different from the control: *p<0.05, **p<0.01, ***p<0.001.

**Figure 4:**
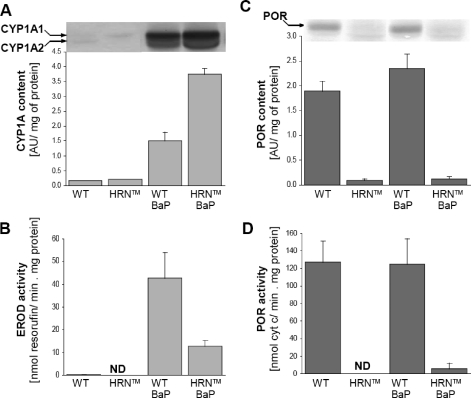
Induction of CYP1A (**A,B**) and POR (**C,D**) protein levels (**A,C**) and activities (**B,D**) in livers of female mice with deleted (HRN™) or intact (WT) liver POR, uninduced or treated (i.p.) with 5 × 125 mg of BaP per kg of body weight. Inset in A: immunoblots of microsomal CYP1A1/2 stained with chicken antibody against rat CYP1A1. Inset in C: immunoblots of microsomal POR stained with chicken antibody against rabbit POR. Microsomes were pooled from livers of 3 animals. Mean and SD evaluated from three separate experiments (n = 3). ND = not detectable at used conditions.

Ellipticine and BaP induced expression of CYP1A1 and 1A2 proteins, which leads to an increase their enzymatic activities in livers of animal models used in the experiments (rats for ellipticine and mice for BaP) (Figures 3 and 4).

As shown in Figure 3, the induction of CYP1A by ellipticine was dose-dependent. The increase in expression of CYP1A proteins correlated with that in specific CYP1A-mediated activity, EROD (Table 1).

**Table 1:** Specific CYP1A activity (EROD) in hepatic microsomes of control and ellipticine-treated rats.

CYP activity	Control rats		Ellipticine-treated rats	
		
Male	Female	Male	Female
EROD	80.7±2.0	225.8±50.5	551.4±92.2	1737.5±161.3

a Each value (pmol of reaction product/min/nmol CYP) represents the mean ± standard deviation of data from two rats in two separate assays (n = 4).

In the case of BaP, mouse models, HRN™ and its parental WT-line, were utilized for the induction experiments. HRN™ mice were found to be more susceptible to BaP-mediated CYP1A induction than the WT mouse line (Figure 4A). Using this model, lacking hepatic POR, we also evaluated whether expression of this enzyme is influenced by treating animals with BaP. Treatment of mice with BaP led to a moderate increase in expression of hepatic POR in both WT (1.2-fold increase) and HRN™ mice (1.4-fold increase). In spite of POR deficiency, CYP1A activity (EROD) was restored by BaP treatment in HRN™ mice, representing the 73-fold increase in EROD activity in microsomes of uninduced WT mice and 30% of this activity in BaP-induced WT mice (Figure 4B).

### DNA adduct formation *in vivo*

**Figure 5:**
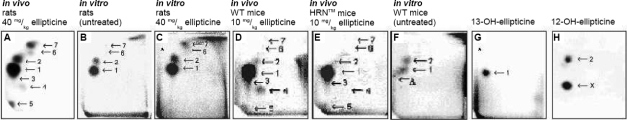
Autoradiographic profile of ellipticine-derived DNA adducts: -*in vivo* in liver DNA of ellipticine-treated rats (**A**); HRN™ (**D**) and WT (**E**) mice. -*in vitro* in calf thymus DNA after ellipticine activation with hepatic microsomes of untreated (**B**) and ellipticine-treated (**C**) male rats and wild-type mice (**F**) -*in vitro* in calf thymus DNA reacted directly with ellipticine metabolites 13-hydroxyellipticine (**G**) or 12-hydroxyellipticine (**H**) (without enzymatic activation). Analyses were performed by the nuclease P1 version of the ^32^P-postlabelling assay.

**Figure 6:**
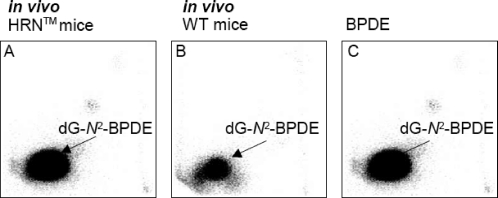
Autoradiographic profiles of BaP-derived DNA adducts *in vivo* in liver DNA of HRN™ (**A**) and WT (**B**) mice treated with 5 times 125 mg of BaP/kg body weight and *in vitro* in salmon testis DNA modified with BPDE (**C**) (without enzymatic activation). Analyses were performed by the nuclease P1 version of the ^32^P-postlabelling assay.

**Figure 7:**
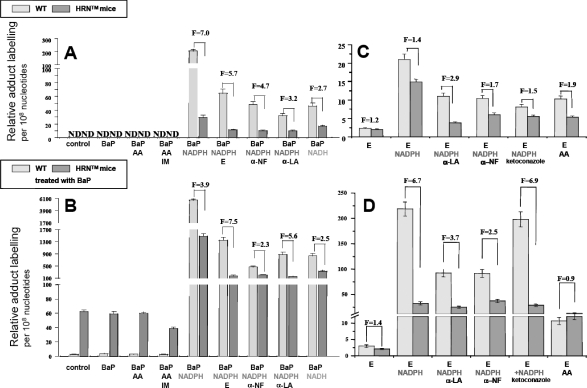
DNA adduct formation after activation of BaP (**A,B**) or ellipticine (**C,D**) with microsomes from livers of HRN™ and WT mice, untreated (**A,C**), or treated with 5 × 125 mg/kg BaP (**B,D**). Each value represents the mean of two separate analyses (n = 2). F = fold increase, WT mice: HRN™ mice; ND = not detected. Control = microsomes + DNA without cofactor; AA = arachidonic acid; IM = indomethacin; E = ellipticine; α-NF = α-naphthoflavone; α-LA = α-lipoic acid.

In further part of the study, we evaluated the potential of ellipticine and BaP to induce DNA adduct formation *in vivo*. Mice were used as models in these experiments. Treatment of individual mouse strains with ellipticine and BaP resulted in DNA adduct formation (Figure 5–7). The livers of all animal models were the major target organ for DNA adduct formation. Comparative analyses on TLC and HPLC have shown that DNA adduct formation *in vivo* proceeds *via* the reactive metabolite BPDE bound to the *N* ^2^ position of guanine (dG-*N* ^2^-BPDE) for BaP (Figures 2 and 6) and *via* 13-hydroxy- and 12-hydroxyellipticine in the case of the two major ellipticine-derived DNA adducts (spots 1 and 2 in Figures 1 and 5).

The experiments employing the mouse models helped us to improve our knowledge on the efficiency of CYPs to activate ellipticine and BaP. Levels of ellipticine-derived adducts in livers of HRN™ mice (figure 5E) were reduced (by up to 65%) relative to levels in WT mice (Figure 5D), indicating that POR-mediated CYP enzyme activity is important for the oxidative activation of ellipticine to metabolites generating these adducts.

In contrast to these results, the highest DNA binding of BaP was observed in livers of HRN™ mice (Figure 6A) which was 6.4-fold (p<0.01) higher than DNA binding in WT mice (Figure 6B). This unexpectable finding indicates increasing the CYP-mediated activation of BaP by lack of POR in the liver.

### Activation of ellipticine and BaP by hepatic microsomes

In order to further investigate the participation of CYPs in activation of ellipticine and BaP and which of these enzymes play the major role, the *in vitro* experiments were carried out. First, incubations of DNA with BaP and/or ellipticine with microsomes isolated from livers of HRN™ and WT mice, untreated or treated with BaP, were performed. In all cases, the patterns of DNA adducts formed by ellipticine and BaP in these experiments were essentially the same as those found *in vivo* (Figures 5 and 6), generated by the pathways shown in Figures 1 and 2. The identity of adducts formed by both compounds *in vitro* with those formed *in vivo* was proved using the TLC and HPLC-cochromatography (data not shown).

Hepatic microsomes isolated from animals treated with ellipticine or BaP were always more effective to form ellipticine- and BaP-derived DNA adducts (Figures 5 and 7) than microsomes from untreated animals. A decrease in levels of ellipticine-derived adducts formed by microsomes from HRN™ mice compared with WT-mice (Figure 7C–D) correlated with almost 2-fold lower levels of 13-hydroxy- and 12-hydroxyellipticine, the metabolites generating the ellipticine-DNA adducts, formed by these microsomes (Stiborová *et al*., 2008).

NADPH-dependent activation of BaP was even 4- to 7-fold lower in HRN™ compared to WT mouse microsomes (Figure 7A–B). The study investigating the pattern of BaP metabolites formed by microsomes from livers of all mouse groups (control HRN™ and WT-mice as well as these mice treated with BaP), which might explain this feature is under way in our laboratory. Preliminary results suggest that the treatment of WT mice with BaP influenced only the relative metabolites ratio instead of the total efficiency of BaP metabolite formation.

In all model systems, the use of POR-inhibitor (α-lipoic acid), CYP1A-inhibitors (α-naphthoflavone, ellipticine) and a CYP3A-inhibitor (ketoconazole) decreased the DNA-adduct formation by both compounds (Figure 7). These results suggest that even very low levels of the POR enzyme in livers of HRN™ mice are still sufficient to mediate CYP-catalyzed activation reactions.

In order to determine which CYPs and/or other enzymes are responsible for DNA adduct formation by both compounds, modulation of microsome-mediated activation with cofactors and inhibitors of individual enzymes was utilized. Addition of NADH, a cofactor of microsomal NADH:cytochrome b_5_ reductase, acting as second electron donor for CYP-dependent systems, lowered the difference between HRN™ and WT microsomal BaP-activation (Figure 7A–B). Arachidonic acid (AA), a cofactor of COX-dependent oxidation, was effective to activate ellipticine to species forming DNA adducts (Figure 7C–D), but not to mediate the BaP-DNA adduct formation (Figure 7A–B). On the contrary, an inhibitor of COX, indomethacin (IM) decreases BaP activation in incubations with hepatic microsomes from BaP-treated HRN™ mice, by 30–40% (Figure 7B). These results may indicate the participation of COX in activation of both compounds, but with lower efficiency than CYPs.

## Discussion

As shown in several studies published previously, ellipticine and BaP are two xenobiotics that react with DNA forming covalent DNA adducts (for a summary, see Stiborová *et al.*, 2006b; Arlt *et al.*, 2008). This genotoxic effect is mediated by their CYP-mediated metabolism. Although the CYP enzymes activating ellipticine and BaP to species binding to DNA *in vitro* have already been identified (Baird *et al*., 2005; Stiborová *et al.,* 2001; 2003a; 2004; 2006a; 2008; Kotrbová *et al.,* 2006; Arlt *et al.,* 2008), the knowledge on the real impact of these CYPs on the activation of these compounds *in vivo* is limited. Likewise, the effects of repeated exposure of organisms to these compounds on enzyme-mediated activation process are scarce.

To evaluate the contribution and importance of hepatic CYP enzymes to the activation of ellipticine and BaP *in vivo,* we have used in our former and present studies the rats and especially the HRN™ mice, lacking POR and thus also POR-mediated CYP enzyme activity in the liver (Henderson *et al*., 2003; 2006), as model organisms. The use of the HRN™ mouse model has already contributed to resolve the *in vivo* enzymatic activation of several environmental toxicants, including carcinogenic 3-nitrobenzanthrone, activated by cytosolic nitroreductases rather than microsomal POR, and its reductive metabolite 3-aminobenzanthrone (Svobodová *et al*., 2007), whose activation is CYP-dependent (Arlt *et al.,* 2003, 2004, 2005, 2006).

Ellipticine and BaP significantly induced expressions of CYP1A1 and 1A2 proteins as well as their enzymatic activity such as EROD in rodent livers. The CYP1A induction resulted in a significant increase in levels of ellipticine- and BaP-derived DNA adducts *in vitro*, in incubations of ellipticine or BaP with microsomes from rats treated with these compounds than in incubations with control microsomes. This is an important finding, because CYP1A enzymes are essential for ellipticine and BaP metabolism. Indeed, the importance of POR-mediated CYP1A1 activation of both compounds *in vitro* was confirmed by inhibition studies using a specific POR inhibitor, α-lipoic acid, and a CYP1A inhibitor, α-naphtoflavone.

Analogously to the results found *in vitro*, the levels of two major DNA adducts in animals treated with ellipticine were significantly decreased in liver DNA of HRN™ mice, confirming the importance of CYP enzymes in ellipticine activation in this organ *in vivo*. Inhibition of NADPH-dependent ellipticine activation in hepatic microsomes of HRN™ and WT mice by α-NF and ketoconazole suggests that CYPs of 1A and 3A subfamilies play a major role in this process in mice livers, analogously to human and rat livers (Stiborová *et al*., 2001; 2003a, b). Nevertheless, the reduction of DNA adduct formation in the liver of HRN™ mice was not absolute, being ~65%. Likewise, the decrease in levels of these two ellipticine-DNA adducts in hepatic microsomes of HRN™ mice caused by inhibitors of POR and CYPs was between 40–65%. These results suggest that other enzymes may also activate ellipticine in mice livers. A potential of arachidonic acid, a cofactor of COX enzymes, to increase the formation of these adducts in mice hepatic microsomes *in vitro* indicate that COX might be one of such enzymes.

On the contrary, BaP-induced DNA adduct formation *in vivo* was significantly increased in liver of HRN™ compared to WT mice, indicating that the real function of POR-mediated CYPs reactions is the BaP detoxification. Taken our results together with those from the CYP1-deletion studies (Uno *et al*., 2004, 2006), there is a remarkable discrepancy between the *in vivo* DNA adduct levels and *in vitro* BaP activation, which is still very difficult to explain. Although hepatic CYP enzyme activity has been essentially inactivated by the conditional deletion of hepatic POR, in non-parenchymal liver cells, the POR deletion may be incomplete. This residual POR activity, in combination with more pronounced induction of CYP enzymes (and probably also by induction of POR) in livers of HRN™ mice liver may explain these *in vitro* findings. Another explanation could be the induction of other metabolizing enzymes.

Taken together, these results show evidence of the crucial role of CYP1A enzymes in ellipticine and BaP genotoxic effect *in vivo.* By inducing CYP1A1/2, both compounds modulate their either pharmacological (ellipticine) and/or genotoxic potential (both chemicals); ellipticine increases its own metabolism leading to enhanced formation of reactive species forming DNA adducts and BaP enhances its detoxification process.
